# A Coal Economizer

**Published:** 1883-01

**Authors:** 


### A COAL-ECONOMIZER.

Mr. Pridgin Teale says truly that our present open fire-places are all on the furnace system, and advocates the adoption of a remarkably simple plan, which converts them at once into slow combustion grates. A plate of iron to inclose the space between the hearth and the lowest bar of the grate is all that is wanted, or, in his own words, "a simple shield resting on the hearth and rising as high as the bottom bar of the grate." It costs two or three shillings, and requires no fixing or "man's time." The economizer makes three tons of coal do the work of four. By its universal use in the Leeds Infirmary it saves £100 a year in coal. It consumes all cinders, and leaves at the bottom of the grate a fine ash, valuable to farmers. It is reckoned that if everybody in the United Kingdom converted his fireplace into a slow-combustion grate, on the principles laid down by Mr. Teale, there would be a saving in the consumption of coal of nearly 9,000,000 tons in the year. Having heard of the economizer a few months ago, I got Jones, of Down street, Piccadilly, to put one into my kitchen stove and drawing-room fireplace, and have found no discontent expressed below, and much satisfaction felt above, as the fire keeps in regardless of much attention from the butler, and always looks cherry and bright when I come home.

